# Development of a simple score to predict outcome for unresponsive wakefulness syndrome

**DOI:** 10.1186/cc13745

**Published:** 2014-02-26

**Authors:** Xiao-gang Kang, Li Li, Dong Wei, Xiao-xia Xu, Rui Zhao, Yun-yun Jing, Ying-ying Su, Li-ze Xiong, Gang Zhao, Wen Jiang

**Affiliations:** Department of Neurology, Xijing Hospital, Fourth Military Medical University, Xi’an, 710032 China; Division of Neurocritical Care, Xuanwu Hospital, Capital Medical University, Beijing, 100053 China; Department of Anaesthesia, Xijing Hospital, Fourth Military Medical University, Xi’an, 710032 China

## Abstract

**Introduction:**

Accurate assessment of prognosis for patients with unresponsive wakefulness syndrome (UWS; formerly vegetative state) may help clinicians and families guide the type and intensity of therapy; however, there is no suitable and accurate means to predict the outcome so far. We aimed to develop a simple bedside scoring system to predict the likelihood of awareness recovery in patients with UWS.

**Methods:**

We prospectively enrolled 56 patients (age range 10 to 73 years) with UWS 3 to 12 weeks post-onset. We collected demographic data and performed neurological, serological and neurophysiological tests at study entry. Each patient received a one year follow-up, during which awareness recovery was assessed by experienced physicians on the basis of clinical criteria. Univariate and multivariable analyses were employed to assess the relationships between predictors and awareness recovery.

**Results:**

A total of 56 participants were included in the study; of these, 24 patients recovered awareness, 3 with moderate disabilities, 8 with severe disabilities, 12 were in a minimally conscious state, and 1 died after recovery. During the study, 23 patients remained in UWS and 9 died in UWS. Motor response, type of brain injury, electroencephalogram reactivity, sleep spindles and N20 were shown to be independent predictors for awareness recovery. Based on their coefficients in the model, we assigned these predictors with 1 point each and created a 5-point score for prediction of awareness recovery. The resulting score showed good predictive accuracy in the derivation cohort. The area under the receiver operating characteristic curve for the score was 0.918 with 87.50% sensitivity.

**Conclusion:**

This simple bedside prognostic score can be used to predict the probability of awareness recovery in UWS, thus provide families and clinicians with useful outcome information.

## Introduction

Advances in intensive care have led to an increase in the number of patients who survive severe brain injury [1, 2]. Although some of these patients recover from coma within the first few days after the insult, many do not, and some evolve to an unresponsive wakefulness syndrome (UWS; previously called vegetative state) [3], which often results in catastrophic familial, economic and social consequences. Thus, accurate outcome prediction of UWS is of paramount significance both for clinicians and families, and may have a major influence on decision making concerning the level of care or services provided [4–6]. However, at present, most prognostic studies have begun in the acute phase of brain injury when the patient is still in coma and the diagnosis of UWS is not yet defined [7–11]. This provides little guidance to clinicians who see patients who have evolved from coma into UWS, and who wish to assess the likelihood of further progress.

Recently, two studies by Estraneo *et al*. directly addressed this issue and they reported that some parameters at the chronic stage, such as pupillary light reflex and N20, could provide useful clues to the outcome prediction for UWS [12, 13]. Nevertheless, no item had a very high predictive specificity [12]. Moreover, up to now, no prognostic model has yet been constructed to predict the probability of awareness recovery in UWS by integrating, quantifying and standardizing multiple predictors.

Accordingly, we performed a clinical prospective study serving two proposes. First, we aimed to identify prognostic markers for awareness recovery in a sample of inpatients who were in UWS during three to twelve weeks post onset. Second, based on identified predictors, we sought to construct a simple bedside score to achieve more reliable outcome prediction for UWS.

## Material and Methods

### Patients

We conducted a prospective longitudinal non-interventional study. From January 2008 to December 2011, we prospectively enrolled all consecutive patients who were admitted to the department of neurology in Xijing hospital, Fourth Military Medical University, one of the largest hospitals in Northwestern China, for the diagnosis of UWS and evaluation of brain function. Patients were eligible for this study if they met standard clinical diagnostic criteria of UWS [14, 15], with the time post-injury longer than three weeks but less than three months. Exclusion criteria were: (1) premorbid history of developmental, psychiatric or neurologic illness resulting in documented functional disabilities up to time of the injury; and (2) severe coexisting systemic disease with a limited life expectancy. The present study was carried out in agreement with Chinese laws and the Helsinki declaration relative to patients’ rights, and was approved by the ethics committee of Xijing hospital. Informed consent was waived, because the study’s design did not involve changes of the usual medical practices.

### Procedures

During the first week after admission, all patients were assessed with the Coma Recovery Scale-Revised (CRS-R) once daily to confirm the diagnosis of UWS. Fifty-six inpatients (41 male and 15 female) fulfilled inclusion and exclusion criteria. Then we systematically collected clinical data, measured serum neuron specific enolase (NSE) level, and performed bedside neurophysiological tests. Once the patients passed the examination and evaluation procedures, they were transferred to some secondary hospitals, where they only received the basic care and medical demands. Some patients occasionally received a short-term acupuncture treatment. During the secondary hospital stay, they were assessed at least once a week by experienced physicians, who had been specifically prompted to search for signs of awareness. All patients were followed up for at least one year after study entry. The study protocol contained no guidelines for withholding or withdrawing treatment.

### Definition of the predictor variables

Based on the results of previous studies, we chose 11 predictor variables possibly associated with recovery from UWS in this study. Four variables concerned the main patient characteristics: 1) age; 2) sex; 3) time from injury to study entry; 4) types of brain injury, coded as traumatic or nontraumatic. Three variables were extracted from clinical examination: 1) papillary light reflexes, coded as present at least unilateral response or bilateral absent; 2) corneal reflexes, coded as present at least unilateral response or bilateral absent; 3) motor responses to painful stimulation, coded as flexion withdrawal of at least one limb, or absent/extensor motor responses.

One serum biomarker (that is, NSE) was measured after study entry. To avoid hemolysis and false-positive test results, samples for NSE measurement were manually handled and transported to a central laboratory. For accurate determination of NSE levels, an automated immunofluorescent assay (Thermo Scientific Brahms NSE Kryptor® Immunoassay Waltham, Massachusetts, USA) was used. Serum NSE cutoff value was defined as 33 ng/mL [7].

Within one week after study entry, each patient received a standard video- electroencephalogram (EEG, Beijing Sun Electronic Technology Co., Ltd., Beijing, China) and somatosensory evoked potential (SEP, XLTEK NEUROMAX 1004, Canada Oakville, Ontario) tests. The video-EEG was performed with 20 scalp electrodes arranged in accordance with the international 10–20 system and was continuously recorded for at least 24 hours to detect sleep spindle, a kind of waveform distinct from the background with a frequency between 12 and 16 Hz, duration between 0.5 and 2 seconds and occurring in the context of EEG activity [16]. EEG reactivity, defined as a change in the frequency or amplitude of the background activity with a precise time-locked correlation to the noxious stimulation [17], was tested by applying pressure to the nail bed of each hand and to the supraorbital nerve above the medial third of the eyebrow. The cortical N20 wave of median nerve SEPs were recorded using standard procedures. We applied four channels: Fpz- C’4/C’3, right Erb's point/left Erb’s point- C’4/C’3, Fpz- Cv and Fpz- right Erb's point/left Erb’s point. SEPs were recorded after median nerve electrical stimulation of the right and left hand. Square-wave pulses with a duration of 0.2 msec at a repetition rate of three pulses per second were used as stimuli to the median nerve at the wrist. Stimulus intensity was 25 to 30 mA. Three neurophysiological variables were considered: 1) EEG reactivity, classified as present when the EEG responded to any one of those stimuli, or absent; 2) sleep spindles, classified as present when typically and easily recognizable 12 to 14 c/second activity appeared, or absent; 3) N20, classified as present when cortical N20 response was recorded on at least one side after left- and right-side median nerve stimulation, or absent. All EEGs and SEPs were independently evaluated by two experienced neuroelectrophysiological specialists (B Jiang and Y Xu) with no knowledge of the clinical data.

### Definition of outcome

The neurological outcome at the study endpoint was assessed by a skilled hospital staff from our research group on at least two different occasions using the five categories of the Glasgow Outcome Scale (GOS) (1, death; 2, permanent vegetative state; 3, severe disability; 4, moderate disability; 5, good recovery) plus an additional category for patients in a minimally conscious state (MCS) [18, 19]. For the purpose of statistical analysis, we defined score 1 and 2 of GOS as no recovery of awareness, and score 3, 4, 5 and MCS as recovery of awareness. Those patients who recovered awareness and then died because of new etiologic events were considered as ‘recovery of awareness’, and the patients who died without recovering awareness were classified as ‘no recovery of awareness’.

### Statistical analyses

First, we performed univariate comparisons for outcome with Fisher exact tests for categorical variables, and t-tests for continuous variables, as needed. Second, a stepwise multivariate logistic regression was used to identify independent outcome predictors among those found to have a *P* <0.05 on univariate analysis. Third, we created a scoring system for outcome prediction in UWS by assigning points to each risk factor by dividing each β-coefficient in the model by the lowest β-coefficient and rounding to the nearest integer. A predictive score was determined for each subject by adding the points of each factor, with higher scores corresponding to a higher likelihood of recovery in UWS. Last, the performance of the predictive score was evaluated by the receiver operating characteristic curve (ROC) analysis. An area under the ROC curve >0.75 is considered consistent with a good discrimination ability. The best cutoff value of the score that predicts the primary end point was determined from the ROC curve.

## Results

A total of 56 UWS patients were enrolled in the study, including 23 patients with traumatic brain injury, 14 patients with cardiac arrest, 10 with carbon monoxide poisoning, 4 with stroke, 3 with apnea, and 2 with drowning. The study cohort was 10- to 73-years old and 73.2% were male. At the endpoint, the outcomes of the 56 patients were as follows: 3 moderate disabilities (5.4%), 8 severe disabilities (14.3%), 12 MCS (21.4%), 23 UWS (41.1%) and 10 deaths (17.9%). One patient recovered awareness but then died because of infectious disease four months post onset; and he was included in the conscious group. Nine patients had died without recovering consciousness during the study period. The causes of death were infectious diseases in four cases, status epilepticus in one case, cerebral hemorrhage in one case and unknown in the four others.

Demographic, clinical, and neurophysiologicalal variables collected in all patients at study entry are shown in Table 1. Univariate analysis indicated that motor response, type of brain injury (BI), EEG reactivity, sleep spindles and N20 differed significantly between patients who recovered and those who did not recover. As shown in Table 2, the five above-mentioned variables had a high specificity for recovery of awareness, but among them only sleep spindles showed a high (70.8%) sensitivity. Although motor response showed the highest specificity (83.3%), its sensitivity was quite low. In general, no item had both a very high sensitivity and specificity.

**Table 1:** **Demographic, clinical, and instrumental features at study entry**

Variable	Awareness (number = 24)	Unawareness (number = 32)	Total (number = 56)	t/ ***χ*** ^2^	***P***
Age, years, mean ± SD	41.17 ± 15.9	39.66 ± 15.8	40.30 ± 15.6	0.353	0.726
Female/male	6/18	9/23	15/41	0.069	0.793
Time post onset, weeks, mean ± SD	6.08 ± 2.5	5.97 ± 2.0	6.02 ± 2.2	0.192	0.849
Type of BI, trauma/nontrauma	15/9	8/24	23/33	8.093	0.004
NSE (μg/L), <33/≥33	19/5	18/14	37/19	3.319	0.068
Pupillary light reflex, yes/no	23/1	31/1	54/2	0.043	0.836
Corneal reflex, yes/no	23/1	29/3	52/4	0.594	0.441
Motor response, flexor/extensor or absent	21/3	17/15	39/17	8.008	0.005
EEG reactivity, present/absent	16/8	8/24	26/30	9.944	0.002
Sleep spindles, present/absent	17/7	7/25	24/32	13.891	0.0002
N20, present/absent	15/9	8/24	23/33	8.0935	0.004

BI, brain injury; NSE, neuron specific enolase; EEG, electroencephalogram.

**Table 2:** **Performance of the variables for recovery of awareness**

Variable	Reference	Sensitivity			Specificity			PPV			NPV		
		Ratio	%	95% CI	Ratio	%	95% CI	Ratio	%	95% CI	Ratio	%	95% CI
Type of BI	Trauma	15/23	65.2	42.8 to 82.8	24/33	72.7	54.2 to 86.1	15/24	62.5	40.8 to 80.4	24/32	75.0	56.2 to 87.9
Motor response	Flexor	21/38	55.3	38.5 to 71.0	15/18	83.3	57.7 to 95.6	21/24	87.5	66.5 to 96.7	15/32	46.9	29.5 to 45.0
EEG reactivity	Present	16/24	66.7	44.7 to 83.6	24/32	75.0	56.2 to 87.9	16/24	66.7	44.7 to 83.6	24/32	75.0	56.2 to 87.9
Sleep spindles	Present	17/24	70.8	48.8 to 86.6	25/32	78.1	59.6 to 90.1	17/24	70.8	48.8 to 86.6	25/32	78.1	59.6 to 90.1
N20	Present	15/23	65.2	42.8 to 82.8	24/33	72.7	54.2 to 86.1	15/24	62.5	40.8 to 80.4	24/32	75.0	52.2 to 87.9

BI, brain injury; CI, confidence interval; EEG, electroencephalogram; NPV, negative predictive value; PPV, positive predictive value.

**Figure 1:**
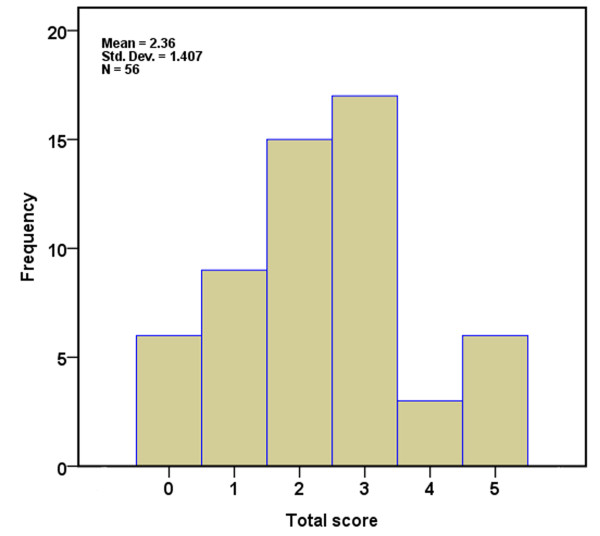
**Distribution of scores for development cohort.**

**Figure 2:**
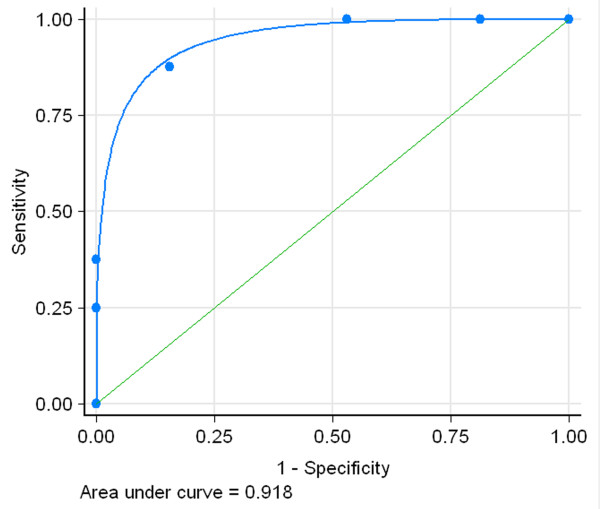
**ROC curve based on the TMSEN score for predicting the recovery of UWS.** ROC, receiver operating curve; UWS, unresponsive wakefulness syndrome.

Next, the five variables were entered into a logistic regression model with recovery of awareness as the outcome. The result showed that all of them were significant independent predictors for recovery of awareness. The coefficients for each predictor are detailed in Table 3. By assigning integer weights to each variable category based on the relative magnitude of the coefficient in the multivariable model, we created a score, which we named TMSEN (type of BI - motor response - sleep spindles - EEG reactivity - N20), to predict the chance of recovery of awareness within one year after study entry. Table 4 shows the allocation of scoring points based on the regression coefficients. These variables were assigned one point each. The prediction score ranged from 0 to 5 and its distribution is shown in Figure 1. The ROC curve for the weighted score showed good discriminant power with an area under curve (AUC) of 0.918 (95% CI, 0.848 to 0.987; Figure 2). The cut-off score with the maximum sum of sensitivity and specificity was a score ≥3. Using the cut-off value, the score had sensitivity of 87.50% with positive predictive value (PPV) of 80.77% and specificity of 84.38% with a negative predictive value (NPV) of 90.0% for predicting awareness recovery (Table 5). The performance of the score was better than any single predictor (Table 2).

**Table 3:** **Multivariate logistic regression analysis of factors associated with recovery within one year after study entry**

Variable	Coefficient	Odds ratio (95% CI)	***P***	Weighted integer coefficient
Type of BI	2.301	9.989(1.351 to 73.839)	0.024	1
Motor response	2.672	14.464(1.355 to 154.367)	0.027	1
EEG reactivity	3.075	21.648(2.212 to 211.870)	0.008	1
Sleep spindles	2.405	11.083(1.795 to 68.441)	0.010	1
N20	2.857	17.404(1.976 to 153.267)	0.010	1

BI, brain injury; CI, confidence interval; EEG, electroencephalogram.

**Table 4:** **Point allocation for TMSEN score based on regression coefficients from the prediction model**

Relative factor	Catagories	Points
Type of BI	Trauma	1
	Nontrauma	0
Motor response	Flexor	1
	Extensor or absent	0
EEG reactivity	Present	1
	Absent	0
Sleep spindles	Present	1
	Absent	0
N20	Present	1
	Bilaterally absent	0
Total		5

BI, brain injury; EEG, electroencephalogram.

**Table 5:** **Performance measures for the scoring system**

TMSEN score	%
Sensitivity	87.50
Specificity	84.38
Positive predictive value	80.77
Negative predictive value	90.00

**Figure 3:**
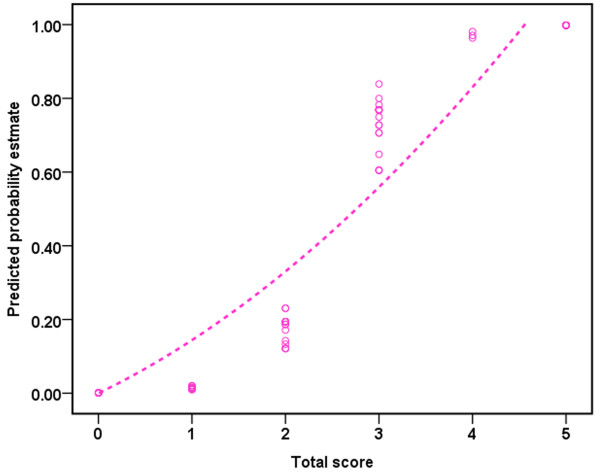
**Points and their corresponding predicted estimates of the recovery of UWS based on the TMSEN score.** UWS, unresponsive wakefulness syndrome.

The new prognostic score provided a desired predictive accuracy. Twenty-one of 24 (87.50%) patients who recovered within one year had a score of 3 or more (sensitivity) and 27 of 32 (84.38%) patients who did not recover within this interval had a score of 0 to 2 (specificity). Taking into account the prevalence of recovery within one year in this population, a score of 3 or more translates into an 80.77% probability of recovery within one year (PPV) whereas a score of 0 to 2 translates into a 90.00% NPV. Figure 3 shows prediction estimates based on the point scoring system and the probability of recovery of awareness within one year increases as the score rises.

## Discussion

This study was undertaken to develop a clinically useful score to predict recovery of awareness for patients with UWS. To maximize clinical use, this prediction score was designed to include bedside, non-invasive, and easily-operated measured factors. The results showed that motor response, type of BI, EEG reactivity, sleep spindles and N20 were significant independent predictors for recovery of awareness at one year after study entry. Based on these predictors and their regression coefficients in the model, we created a simple bedside five-point score, TMSEN, to predict the probability of recovery in UWS. The TMSEN score showed good predictive accuracy in the derivation cohort and was able to discriminate among patients who recovered awareness within one year after study entry and those who did not.

This score about prognostic information of UWS may be of value for patients and the health care system. First, widespread media attention to UWS care has increased public awareness of the importance of these issues. Second, efforts to control rising health care costs have provided hospitals, managed care organizations and payers increasing incentives to allocate expenditures more efficiently for patients likely to be close to the end of life. In this study, we first employed statistical methods to derive a predictive assessment tool to identify, quantify and characterize the recovery of patients with UWS. Thus, this study should be seen as an important step toward the study prognosis in patients with UWS.

Previous studies have shown that the variables including motor response to pain, EEG reactivity, sleep spindles and N20 are important predictors for the outcome of comatose patients with severe brain injury when measured in the acute phase [8, 20–29]. Our study further confirmed that these variables, even if measured in the late phase when patients have evolved into UWS, can predict the outcome. Likewise, we found that the outcome in posttraumatic UWS is better than in nontraumatic UWS as in previous studies [13, 30]. Given that the immature brain is generally more plastic than the mature brain and that the brains of children are better able to adapt and recover from injury than the brains of adults [31], we chose age as a predictor variable. Nevertheless, we did not find a significant relationship between age and outcome in our sample, which seems to conflict with the findings reported by Luauté *et al*. [18]. This could be explained by the fact that there is no direct relationship between age and the severity of clinical conditions, and the latter has an important influence on patients’ recovery. However, a further larger sample study and stratification analysis are needed to clarify this point.

In agreement with the previous study [18], we did not find a significant relationship between recovery and brainstem reflexes (pupillary light reflex and corneal reflex). It is likely that the brainstem function is preserved in UWS [32]. We also failed to identify NSE as an independent predictor. NSE is found almost exclusively in neurons and cells of neuroendocrine origin, which is measurable in blood and cerebrospinal fluid. Previous studies have demonstrated that serum NSE levels often reflect the extent of brain injury, and that increased serum NSE within the first three days after cardiac arrest is associated with poor outcome [33–35]. However, serum NSE levels after brain injury displayed a gradual decline over time [36], which indicates that NSE collected from patients in the late phase of brain injury cannot be used as a prognostic factor to predict the outcome of patients with UWS.

Given that the patients in MCS show inconstant but reproducible signs of awareness and generally more favorable outcome than those in UWS [18], we classified them into the recovery group in this study. In total, recovery of awareness was detected in 24/56 patients at the study endpoint, albeit most of them with poor functional outcome. Actually, it is a difficult task to detect awareness in patients who seem to be in UWS, especially to distinguish MCS from UWS. It is well known that consciousness is not an all-or-nothing phenomenon and its clinical assessment relies on inferences made from responses to external stimuli that are observed at the time of the examination [37], which often results in an alarmingly high rate of misdiagnosis of UWS [38, 39]. Accordingly, careful and repeated behavioral assessment is particularly important given that the patients often failed to show any signs of non-reflexive behavior on some occasions but they showed reproducible, but inconsistent, response to some commands (that is, ‘move your leg’) on other occasions. Moreover, there is a small proportion of patients who meet all the behavioral criteria for UWS, but still retain a level of covert awareness that can only be detected by functional magnetic resonance imaging (fMRI) or EEG technology [40, 41]. Cruse *et al*. used four to five assessments with CRS-R to establish the diagnosis at the study entry [41]. However, it is difficult to assess the patients with optimal frequency at the endpoint of follow-up study. In this study, all patients were assessed with the CRS-R once a day in the first week after admission. At study endpoint, we found 23 patients still in UWS based on behavioral assessment on at least two different occasions. However, due to a perhaps suboptimal frequency of behavioral assessment, as well as methodological limitations, the possibility of misdiagnosis of MCS as UWS may exist.

Our study possesses several strengths. First, the five-point score is simple and can be easily memorized, including only five items with one point each: type of BI, motor response, sleep spindles, EEG reactivity and N20. Second, the score incorporating multiple independent risk factors was highly successful in predicting the probability of recovering of awareness in UWS. The score predicts the recovery of awareness in UWS with a sensitivity of 87.50% and a specificity of 84.38%. It is statistically superior in performance to the models using a single variable whether clinical or neurophysiological. Lastly, our study used simple, bedside and non-invasive measured factors for the development of the prediction score. Therefore, we provide an easy tool for clinical practice.

In addition, the present study has some limitations that need to be mentioned. First, the score was developed just at a single site in a university hospital setting, and the development sample was relatively small. Thus, it may not generalize to other settings or populations. Prospective validation in different settings and with other patient populations is needed. Second, the predictors included in this study are not exhaustive, and we omitted several known and suggested variables such as fMRI [40, 42, 43] and positron emission tomography (PET) [44, 45]. Given the issues of expense and accessibility, we were unable to include these variables in the analysis. However, future refinements of this score should consider a role for these predictors. Third, the level and intensity of the care and rehabilitation program for UWS in different secondary hospitals may be not uniform, which could affect the outcome of patients to some extent. Lastly, it would be optimal to validate this score in a large external observational cohort. However, most of the studies of UWS are small, retrospective in nature, and assess relatively few predictors. We failed to identify a published data set with sufficient UWS cases, which assessed all the predictor variables identified in this model.

## Conclusions

We developed a simple bedside prognostic score (TMSEN) consisting of five items: type of BI, motor response, sleep spindles, EEG reactivity and N20, which will provide families and clinicians with useful outcome information. However, it should be recognized that the score simply predicts the probability of recovery, with no judgment being made as to whether the patients continue to receive intensive treatment or not.

## Key messages

- The results of this single-center study show that type of BI, motor response, sleep spindles, EEG reactivity and N20 are independent predictors for awareness recovery in UWS.
- The score incorporating the five above-mentioned independent factors was highly successful in predicting the probability of awareness recovery in UWS. It is statistically superior in performance to any single variable whether clinical or neurophysiological.
- Our study used bedside, non-invasive and easily-operated measured factors for the development of the prediction score. Therefore, we provide an easy assessment tool for the outcome prediction in UWS.
- The prognostic score will provide families and clinicians with useful outcome information, but no judgment could be made on the basis of the score as to whether the patients continue to receive intensive treatment or not.
- Additional larger prospective studies are needed to confirm whether the simple bedside prognostic score may be a helpful tool for clinical practice.

## Abbreviations

AUC: area under the curve
BI: brain injury
CI: confidence interval
CRS-R: Coma Recovery Scale-revised
EEG: electroencephalogram
fMRI: functional magnetic resonance imaging
GOS: Glasgow Outcome Scale
MCS: minimally conscious state
NPV: negative predictive value
NSE: neuron-specific enolase
PPV: positive predictive value
ROC: receiver operating characteristic curve
SEPs: somatosensory evoked potentials
UWS: unresponsive wakefulness syndrome.
